# NSUN4 Is a Dual Function Mitochondrial Protein Required for Both Methylation of 12S rRNA and Coordination of Mitoribosomal Assembly

**DOI:** 10.1371/journal.pgen.1004110

**Published:** 2014-02-06

**Authors:** Metodi Dimitrov Metodiev, Henrik Spåhr, Paola Loguercio Polosa, Caroline Meharg, Christian Becker, Janine Altmueller, Bianca Habermann, Nils-Göran Larsson, Benedetta Ruzzenente

**Affiliations:** 1 Max Planck Institute for Biology of Ageing, Cologne, Germany; 2 Department of Biosciences, Biotechnologies and Biopharmaceutics, University of Bari Aldo Moro, Bari, Italy; 3 Institute for Global Food Security, David Keir Building, Queen's University, Belfast, Northern Ireland; 4 Cologne Center for Genomics, University of Cologne, Cologne, Germany; 5 Department of Laboratory Medicine, Karolinska Institutet, Stockholm, Sweden; Stanford University School of Medicine, United States of America

## Abstract

Biogenesis of mammalian mitochondrial ribosomes requires a concerted maturation of both the small (SSU) and large subunit (LSU). We demonstrate here that the m^5^C methyltransferase NSUN4, which forms a complex with MTERF4, is essential in mitochondrial ribosomal biogenesis as mitochondrial translation is abolished in conditional *Nsun4* mouse knockouts. Deep sequencing of bisulfite-treated RNA shows that NSUN4 methylates cytosine 911 in 12S rRNA (m5C911) of the SSU. Surprisingly, NSUN4 does not need MTERF4 to generate this modification. Instead, the NSUN4/MTERF4 complex is required to assemble the SSU and LSU to form a monosome. NSUN4 is thus a dual function protein, which on the one hand is needed for 12S rRNA methylation and, on the other hand interacts with MTERF4 to facilitate monosome assembly. The presented data suggest that NSUN4 has a key role in controlling a final step in ribosome biogenesis to ensure that only the mature SSU and LSU are assembled.

## Author Summary

Mitochondria perform a number of essential functions in the cell, including synthesis of ATP via the oxidative phosphorylation (OXPHOS) system. Normal mitochondrial function requires coordinated expression of two genomes: mitochondria's own genome (mtDNA), which encodes 13 respiratory chain subunits with essential structural and functional roles for the OXPHOS system, and the nuclear genome encoding the remaining ∼80 subunits. The mtDNA-encoded polypeptides are synthesized on mitochondrial ribosomes (mitoribosomes) located in the mitochondrial matrix. Biogenesis, maintenance and regulation of the complex mitochondrial translation apparatus are poorly understood despite its fundamental importance for cellular energy homeostasis. Here, we show that inactivation of the *Nsun4* gene, encoding a mitochondrial m^5^C-methyltransferase, causes embryonic lethality, whereas tissue-specific disruption of *Nsun4* in the heart causes cardiomyopathy with mitochondrial dysfunction. By performing sequencing of bisulfite-treated RNA we report that NSUN4 methylates C911 in 12S rRNA of the small ribosomal subunit. Surprisingly, NSUN4 can on its own perform this rRNA modification, whereas interaction with its partner protein MTERF4 is required for assembly of functional ribosomes. NSUN4 thus has dual roles in ribosome maturation and performs an important final quality control step to ensure that only mature mitoribosomal subunits are assembled into functional ribosomes.

## Introduction

Expression of mtDNA is essential for production of ATP through oxidative phosphorylation in all eukaryotes. Mammalian mtDNA encodes 13 polypeptides that are translated on mitochondrial ribosomes (mitoribosomes) and are essential for oxidative phosphorylation. The mammalian mitoribosomes are composed of the mtDNA-encoded 12S and 16S rRNAs and more than a hundred different nucleus-encoded ribosomal proteins [1], [2]. In bacteria, rRNA modifications are established at precise points of ribosomal assembly and can participate in rRNA processing events as well as in folding and interactions with neighboring proteins [3]. Modifications of rRNA have also been reported in mammalian mitochondria and the best characterized example is the TFB1M-mediated dimethylation of two highly conserved adenosines at the 3′-end of mitochondrial 12S rRNA, which is necessary for assembly of the SSU [4]. Other putative methyltransferases, such as RNMTL1, MRM1 and MRM2, were recently proposed to play an important role for mitochondrial rRNA methylation, but the residues which they modify remain unknown [5]. Studies of hamster mitochondria have shown that the SSU rRNA, in addition to the above-mentioned two dimethylated adenosines (m^6^_2_A), also contains two cytosine methylations (m^4^C and m^5^C) and one methylated uracil (m^5^U), whereas the LSU rRNA carries three ribose methylated nucleotides, one Um and two Gm [6]. The exact role of each of the modified residues is unclear, but it is worth noting that modified residues tend to concentrate in functionally important regions of ribosomes [7].

We have recently identified a novel mitochondrial rRNA methyltransferase, denoted NSUN4, which belongs to the same family of m^5^C-methyltransferases as the bacterial enzymes RsmB, RsmF and YccW [8]–[10]. In bacteria, RsmB establishes m^5^C-methylation on C967 of 16S rRNA, RsmF methylates C1400, C1404 and C1407 of 16S rRNA and YccW is responsible for m^5^C-methylation on C1962 in 23S rRNA [11]–[14]. The mitochondrial NSUN4 protein, unlike its bacterial counterparts, lacks PUA- or other types of known RNA-interaction domains. Instead, MTERF4 has been shown to form a stable complex with NSUN4 and target this complex to the LSU of the mitoribosome [8]. Inactivation of the *Mterf4* gene leads to inhibition of mitochondrial translation and NSUN4 is no longer targeted to the LSU [8]. Moreover, in the absence of MTERF4, the SSU and LSU are present at increased levels but there is no formation of mature mitoribosomes [8]. The crystal structure of the NSUN4/MTERF4 heterodimeric complex has shown that the very stable interaction between both subunits occurs at the carboxy-terminus of MTERF4 and that both MTERF4 and NSUN4 likely are involved in RNA-binding [9], [10]. It is interesting to note that MTERF3, another member of the MTERF family, recently was reported to have an essential role in controlling the assembly of the LSU of the mitoribosome [15]. The remaining two members of the mammalian MTERF family, MTERF1 [16]–[19] and MTERF2 [20], do not seem to be directly involved in mitoribosomal biogenesis, but rather have roles in regulating mitochondrial transcription.

To gain novel molecular insights into the role of NSUN4 in mitoribosomal assembly, we inactivated the *Nsun4* gene in the mouse. A germline knockout is embryonic lethal and conditional inactivation of *Nsun4* in heart leads to respiratory chain deficiency due to impaired assembly of the mitoribosome with accompanying inhibition of mitochondrial translation. Mapping of m^5^C residues in 12S and 16S rRNA by sequencing of cDNA generated from bisulfite treated RNA showed that there is a single C5-methylated cytosine at relative position 911 in 12S rRNA in wild-type mice that is lost in *Nsun4* knockout mice. Surprisingly, this methylated C911 modification is present in in *Mterf4* mutant mice. Our findings thus show that NSUN4 modifies 12S rRNA at position 911 and this modification requires neither its interaction with MTERF4 nor its targeting to the LSU. In contrast, the NSUN4/MTERF4 complex plays an essential role in LSU assembly or maturation independent of the methylation activity of NSUN4. Indeed, using PAR-CLIP we identified two regions of 16S rRNA, which were crosslinked to MTERF4. The presence of a bi-functional NSUN4 protein involved in methylation of 12S rRNA of the SSU and assembly of the mature SSU and LSU establishes a novel mechanism for coordinated maturation of both ribosomal subunits during formation of translation competent mitochondrial ribosomes.

## Results

### *Nsun4* is essential for embryonic development in the mouse

**Figure 1:**
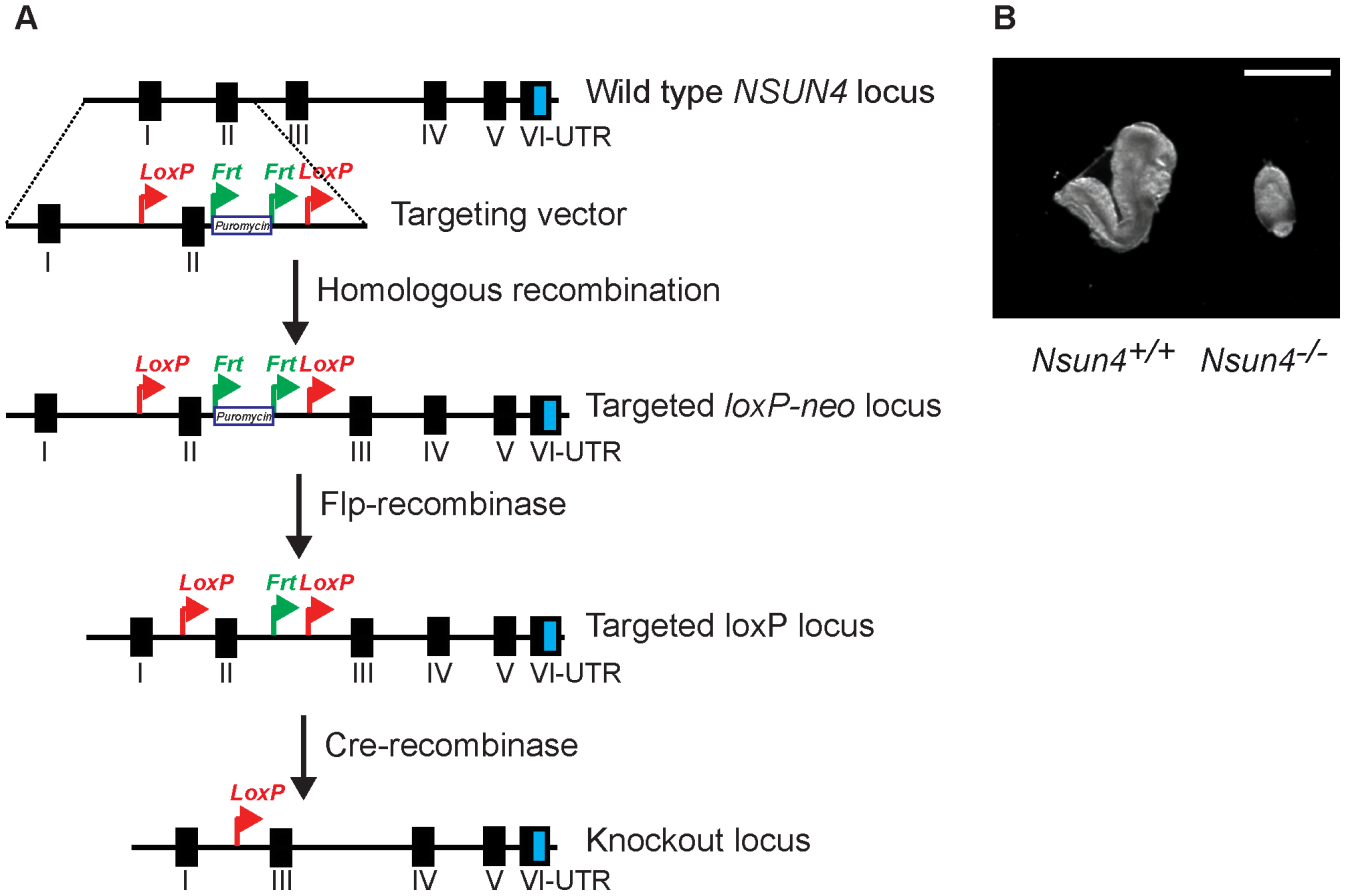
Conditional inactivation of the *Nsun4* gene in the germline. A. Schematic representation of the targeting strategy for disruption of the *Nsun4* gene. Exon II was flanked by loxP-sites. The puromycin resistance gene is flanked by Frt-recombination sites and was used for ES cell selection. Mice with an Frt-flanked puromycin gene were crossed with mice with ubiquitous expression of Flp-recombinase to remove the Puromycin resistance gene. Transgenic mice expressing cre-recombinase were used for breeding with animals with a loxP-flanked *Nsun4* gene to disrupt *Nsun4*. B. Morphological comparison between wild type and whole-body *Nsun4* knockout embryos at E∼8.5. Scale bar = 1 mm.

We generated mice carrying a conditional knockout allele of the *Nsun4* gene to determine the function of NSUN4 in mitochondria (Figure 1A). *Nsun4^loxP/+^* mice were mated with mice expressing cre-recombinase under the control of the *β-actin* promoter in order to obtain germline heterozygous knockout mice (*Nsun4^+/−^*). Intercrossing of *Nsun4^+/−^* mice did not result in any viable *Nsun4^−/−^* mice, whereas *Nsun4^+/+^* and *Nsun4^+/−^* mice were recovered in mendelian proportions, consistent with embryonic lethality. Disruption of essential mitochondrial genes is frequently associated with embryonic lethality at ∼E8.5 [4], [8], [21], [22] and we therefore proceeded with analysis of embryos at this stage. Mutant embryos (*Nsun4^−/−^*) exhibited severely retarded growth with no clearly discernible anatomical structures at E8.5, whereas embryos of the other genotypes appeared normal (Figure 1B). These findings show that NSUN4 has an essential in vivo role.

### Heart specific knockout of *Nsun4* leads to mitochondrial dysfunction

**Figure 2:**
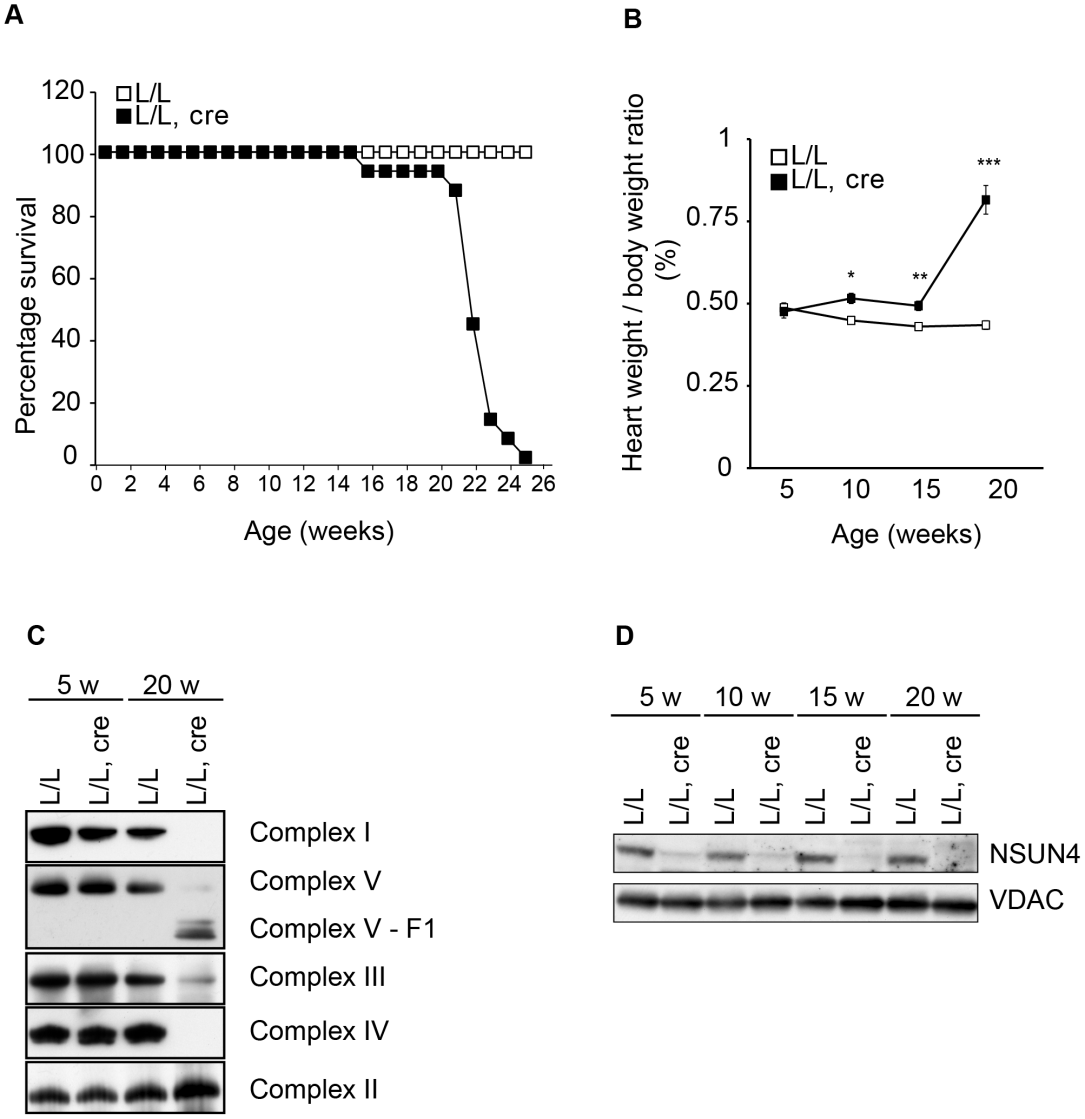
Conditional inactivation of the *Nsun4* gene in heart and skeletal muscle. A. Decreased lifespan of mice with a muscle-specific knockout of *Nsun4*. Survival curve for control (L/L; n = 20; open squares) and mutant mice (L/L, cre; n = 16; filled squares). B. Heart- to body-weight ratio in control (L/L; open squares) and mutant mice (L/L, cre; filled squares). Number of analyzed animals at 5 weeks L/L n = 6, L/L, cre n = 6; at 10 weeks L/L n = 7, L/L, cre n = 8; at 15 weeks L/L n = 5, L/L, cre n = 7; at 20 weeks L/L n = 12, L/L, cre n = 12. Data are represented as mean +/− SEM. *, p<0.05; **, p<0.01; ***p<0.001. Student's t test. C. BN-PAGE analysis of levels of assembled respiratory chain complexes in control (L/L) and knockout (L/L, cre) mice at different ages. D. Western immunoblotting of steady-state levels of NSUN4 in heart mitochondrial extracts from control (L/L) and knockout (L/L, cre) mice at different ages. VDAC was used as a loading control.

We next proceeded with inactivating the *Nsun4* gene in skeletal muscle and cardiomyocytes by crossing *Nsun4^loxP/loxP^* mice with mice expressing cre-recombinase under the control of a creatinine kinase promoter (*Ckmm-cre*). These conditional knockout mice were viable at birth, but had a much shorter life span than control mice with death before the age of 25 weeks (Figure 2A). We determined the heart to body weight ratio and found a gradual increase with age in the tissue-specific knockout mice (*Nsun4^loxP/loxP^*, *+/Ckmm-Cre*), but not in control mice (*Nsun4^loxP/loxP^*) (Figure 2B). Such a progressive cardiomyopathy is commonly seen in mice with heart- and muscle-specific disruption of genes that are essential for oxidative phosphorylation and precedes any detectable phenotypes in skeletal muscle [4], [8], [21], [22]. To assess oxidative phosphorylation capacity in heart, we analyzed the assembly of the five OXPHOS complexes using blue-native PAGE (BN-PAGE) of mitochondrial extracts from control and mutant mice (Figure 2C). This analysis showed that the steady-state levels of assembled OXPHOS complexes were unchanged in 5 weeks-old mice, but were drastically reduced in hearts from 20 weeks-old mutant mice. All complexes containing mtDNA-encoded polypeptides were reduced in abundance, whereas the steady state-levels of complex II, which does not contain any mitochondrially encoded subunits, were unchanged in knockout mice (Figure 2C). Thus, knockout of *Nsun4* in the heart causes mitochondrial dysfunction due to impaired biogenesis of the respiratory chain complexes. Western blot analyzes showed drastically decreased levels of NSUN4 in extracts of heart mitochondria already in 5 weeks-old knockout mice (Figure 2D), consistent with efficient knockout of the *Nsun4* gene.

### Mitochondrial transcription is increased in *Nsun4*-knockout hearts

Mitochondrial dysfunction in the heart triggers a compensatory increase in mitochondrial mass, which often is accompanied by an increase in the steady-state levels of mtDNA and an increase in mitochondrial transcription [4], [8], [15], [21]. We determined mtDNA levels with semi-quantitative TaqMan PCR analysis and found a moderate increase in the steady-state levels in 20 weeks old mutant mice compared to age-matched controls (Figure S1A). This observation was corroborated by the finding of an increase in the steady-state levels of mitochondrial transcription factor A (TFAM), which typically varies with the levels of mtDNA [4], on western blot analysis (Figure S2B).

Next, we examined the steady-state levels of mtDNA-encoded transcripts using northern blotting and autoradiography (Figure S1B, C). All tested mitochondrial transcripts (rRNAs, tRNAs and mRNAs) were significantly increased in mutant mice (Figure S1B, C) consistent with a general upregulation of mitochondrial transcription. Indeed, pulse-labeling of newly synthesized transcripts in isolated mitochondria, followed by autoradiographic analysis of synthesized transcripts, revealed a strong increase in mitochondrial *de novo* transcription in mutant mice compared to controls (Figure S2A). Consistent with these data, western blot analyses showed an increase in the steady-state levels of the mitochondrial transcription factor TFB2M and TFAM in mitochondrial extracts (Figure S2B). Additionally, mutant mitochondria had increased steady-state levels of LRPPRC (Figure S2C), a stability factor for mitochondrial mRNAs, whose steady-state levels correlate with the levels of mitochondrial mRNAs [22]. Thus, knockout of *Nsun4* in the heart leads to increased *de novo* synthesis and increased steady-state levels of mitochondrial transcripts.

### Mitoribosome assembly and mitochondrial translation are inhibited in the absence of NSUN4

**Figure 3:**
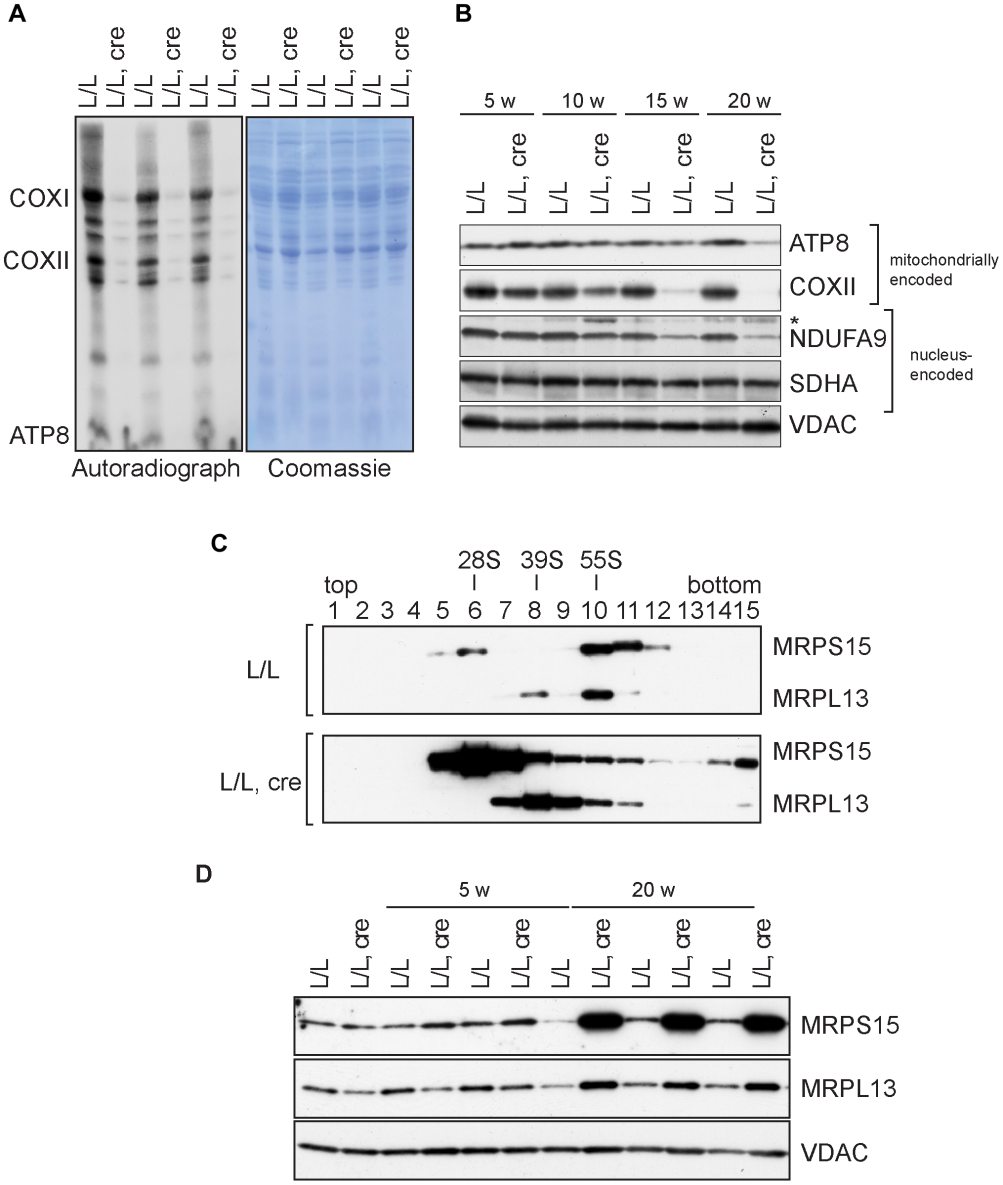
Mitochondrial translation and ribosome assembly in *Nsun4* knockout hearts. A. Pulse-labeling of mitochondrial translation products in isolated heart mitochondria from 20 weeks-old control (L/L) and knockout mice (L/L, cre). The Coomassie-stained gel is a loading control. Known mitochondrial polypeptides are indicated. B. Western blot analysis of steady-state levels of mitochondrially and nucleus-encoded OXPHOS proteins in mitochondria from control and knockout hearts at different ages. VDAC was used as a loading control. *, cross reaction. C. Analysis of mitoribosomal assembly by sucrose gradient ultracentrifugation of heart mitochondrial extracts from control (L/L) and mutant (L/L, cre) mice. Sedimentation of 28S (SSU, fraction 6), 39S (LSU, fraction 8) and 55S (assembled ribosomes, fraction 10) was determined by western blot analysis using MRPS15- and MRPL13-specific antibodies. D. Western blot analysis of steady-state levels of MRPL13 and MRPS15 in heart mitochondrial extracts from control (L/L) and knockout (L/L, cre) mice at different ages. VDAC was used as a loading control.

We have previously shown that NSUN4 is targeted to the LSU through its physical interaction with MTERF4 and that absence of MTERF4 leads to defective ribosomal assembly and inhibition of mitochondrial translation [8]. We therefore proceeded to determine if mitochondrial translation also was inhibited in *Nsun4*-knockout hearts. *De novo* labeling of mitochondrial translation products, followed by gel electrophoresis and autoradiography, revealed a strong inhibition of translation in mutant mitochondria (Figure 3A). Consistent with this, the steady-state levels of the mitochondrially encoded ATP8 and COXII proteins were decreased in knockout hearts (Figure 3B).

Next, we analyzed the assembly of the mitoribosome by ultracentrifugation of control and mutant mitochondrial extracts through linear-density sucrose gradients (Figure 3C). Sedimentation of the SSU and LSU was determined by immunological detection of the protein markers MRPS15 and MRPL13, respectively. In control extracts, we observed a typical sedimentation pattern of the separate SSU (28S) and LSU (39S) as well as of co-sedimentation of both subunits in fully assembled (55S) ribosomes [4], [8], [22]. In contrast, knockout mice exhibited an accumulation of assembled SSU and LSU without a corresponding increase in assembled ribosomes (Figure 3C). Furthermore, the steady-state levels of proteins from the LSU and SSU were increased in knockout mice (Figure 3D). We have previously reported that when ribosomal assembly and/or function are not inhibited, an accumulation of both the SSU and LSU coincides with accumulation of assembled ribosomes [22]. The data we present here therefore indicate that lack of NSUN4 inhibits the association between the SSU and LSU to form functional ribosomes.

### NSUN4 methylates C911 in 12S rRNA

**Figure 4:**
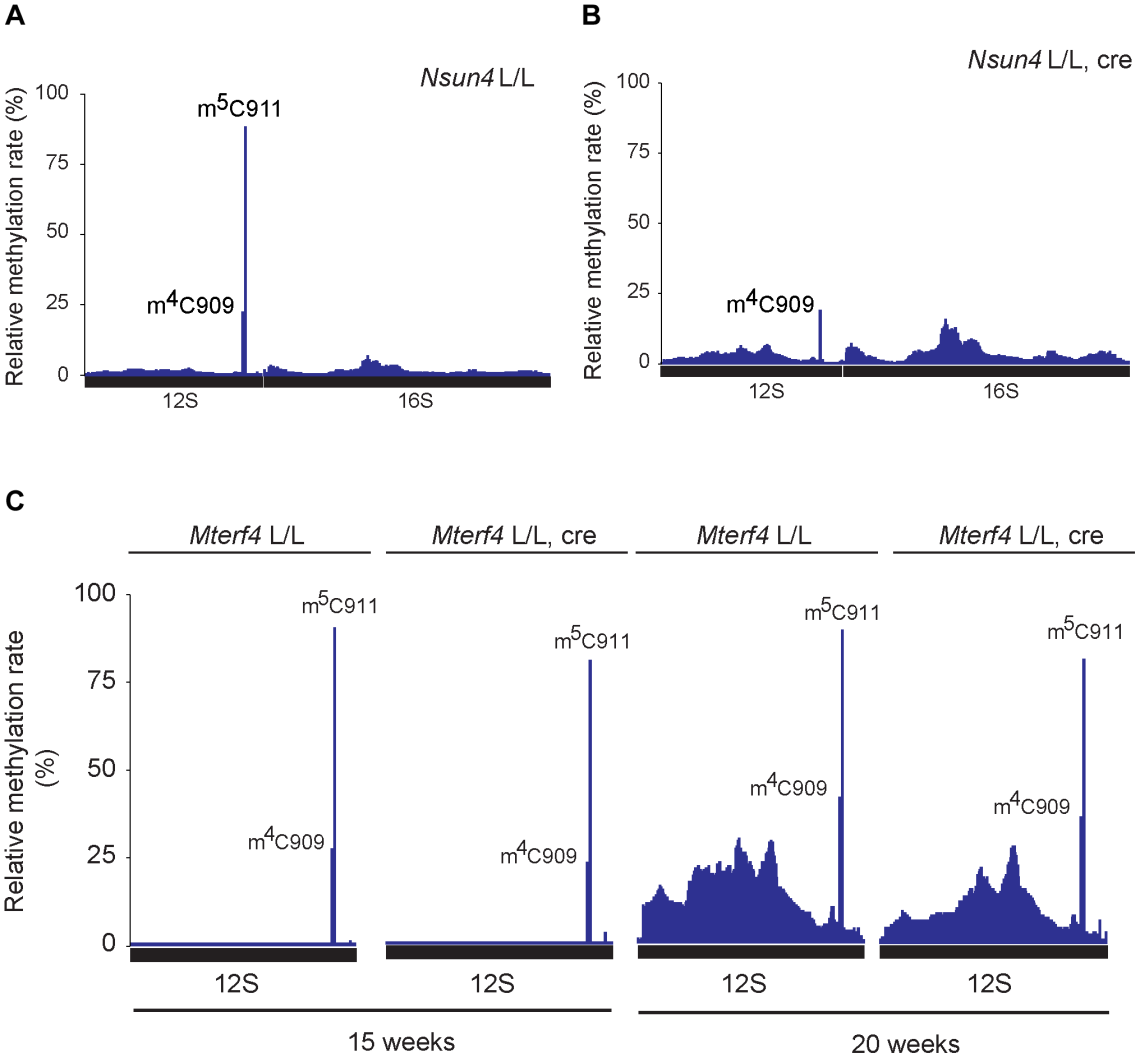
Analysis of rRNA methylation in control, *Nsun4* and *Mterf4* heart tissue specific knockout. A. Relative methylation levels of 12S and 16S rRNA determined after sequencing of cDNA obtained from bisulfite treated RNA from heart mitochondria of control (L/L; n = 3) mice at age 20 weeks. Nucleotide numbers are relative to the 5′-end of the mouse mtDNA gene for tRNA^Phe^. B. Relative methylation levels of 12S and 16S rRNA in NSUN4 knockout (L/L, cre; n = 3) at age 20 weeks. Analysis performed as in panel a. C. Relative methylation levels of 12S rRNA in control (N = 1) and MTERF4 knockout (N = 2) at age 15 weeks and in control (N = 1) and MTERF4 knockout (N = 2) at age 20 weeks.

Previous reports have shown that NSUN4 is an m^5^C-methyltransferase proposed to methylate an unknown residue in 16S rRNA [8]–[10]. However, studies on hamster mitochondria have shown that the 17S rRNA of the LSU does not contain any C5-methylated residues, unlike 13S rRNA of the SSU, which contains one m^4^C and one m^5^C at relative position 911 and 913, respectively (relative to tRNA^Phe^) [6]. We revisited these data by attempting to map all m^5^C residues in mouse 12S and 16S rRNA by using sequencing of cDNA generated from bisulfite treated RNA. The bisulfite method is based on the chemical conversion of non-methylated cytosine to uracil, whereas m^5^C residues are resistant to this treatment and remain unchanged in the treated RNA [23]. Other modifications, like m^4^C can also be detected, but with a lower frequency. Using this approach we probed the methylation status of heart mitochondrial rRNA from control mice (Figure 4A) and detected a single C5-methylated cytosine residue at position 911 in 12S rRNA (methylation rate 87.5±0.9%). Next, we assessed the methylation status of heart mitochondrial rRNA from *Nsun4* knockout mice and found that methylation on C911 was now essentially absent (methylation rate 2.5±2.4%; Figure 4B). Alignment of the mouse and hamster sequences revealed that m^5^C911 in mouse 12S rRNA corresponds to the previously reported m^5^C913 in hamster 13S rRNA (Figure S3A). Thus, NSUN4 methylates C911 in 12S rRNA, and this modification is likely the only m^5^C-methylation present in mammalian mitochondrial rRNAs.

In addition to m^5^C911, there was also a cytosine at position 909 (C909) in mouse 12S rRNA that showed partial resistance to bisulfite treatment (methylation rate 23.6±1.7%; Figure 4A). The corresponding nucleotide in hamster 13S rRNA has previously been reported to harbor an m^4^-methylation [6]. The m^4^C909 modification in mouse 12S rRNA was not affected (methylation rate 21.1±3.4%) in the absence of NSUN4 (Figure 4B), thus showing that the m^5^C911 modification is specifically generated by NSUN4. Additionally, we tested if dimethylation of the highly conserved adenosines A1006 and A1007 (m^6^_2_A1006 and m^6^_2_A1007) in 12S rRNA is affected in the absence of NSUN4. The two m^6^_2_-methylations are established by TFB1M during the maturation of the SSU [4]. Assembly defects of the mitoribosomal SSU, resulting from loss of m^5^C, could possibly affect these adenosine methylation modifications. However, primer extension assays on RNA from control and mutant hearts showed that methylation of these adenosine residues in *Nsun4* knockout mice was indistinguishable from controls (Figure S3B, C).

We proceeded to test if presence of MTERF4 was necessary for methylation of 12S rRNA in vivo by deep sequencing of bisulfite treated RNA from mice with a tissue-specific knockout of *Mterf4* at different ages [8]. In the absence of MTERF4, C911 exhibited methylation rates similar to those in control mice (Figure 4C) showing that interaction between NSUN4 and MTERF4 or targeting of the NSUN4/MTERF4 complex to LSU are dispensable for methylation of C911 in 12S rRNA. Moreover the monosome assembly defect observed in MTERF4 mutant mice cannot be explained by lack of m5C-methylation. Instead, the NSUN4/MTERF4 complex must play another role in LSU assembly.

In view of the finding that NSUN4 can methylate 12S rRNA in the absence of MTERF4, we investigated whether an assembled LSU was needed for methylation of C911 of 12S rRNA. To this end, we performed deep sequencing of bisulfite-treated RNA isolated from *Mterf3-*knockout mouse hearts, which were previously shown to lack an assembled LSU [15]. Methylation of C911 in Mterf3 mutant mice was only mildly reduced in comparison with controls, thus indicating that methylation of 12S rRNA can occur independently of the presence of an assembled LSU (Figure S4). Taken together our data indicate that methylation of 12S rRNA can occur independently of the targeting of NSUN4 to the LSU and that it represents a modification pathway for ensuring proper SSU maturation.

### MTERF4 and NSUN4 preferentially bind double-stranded RNA

**Figure 5:**
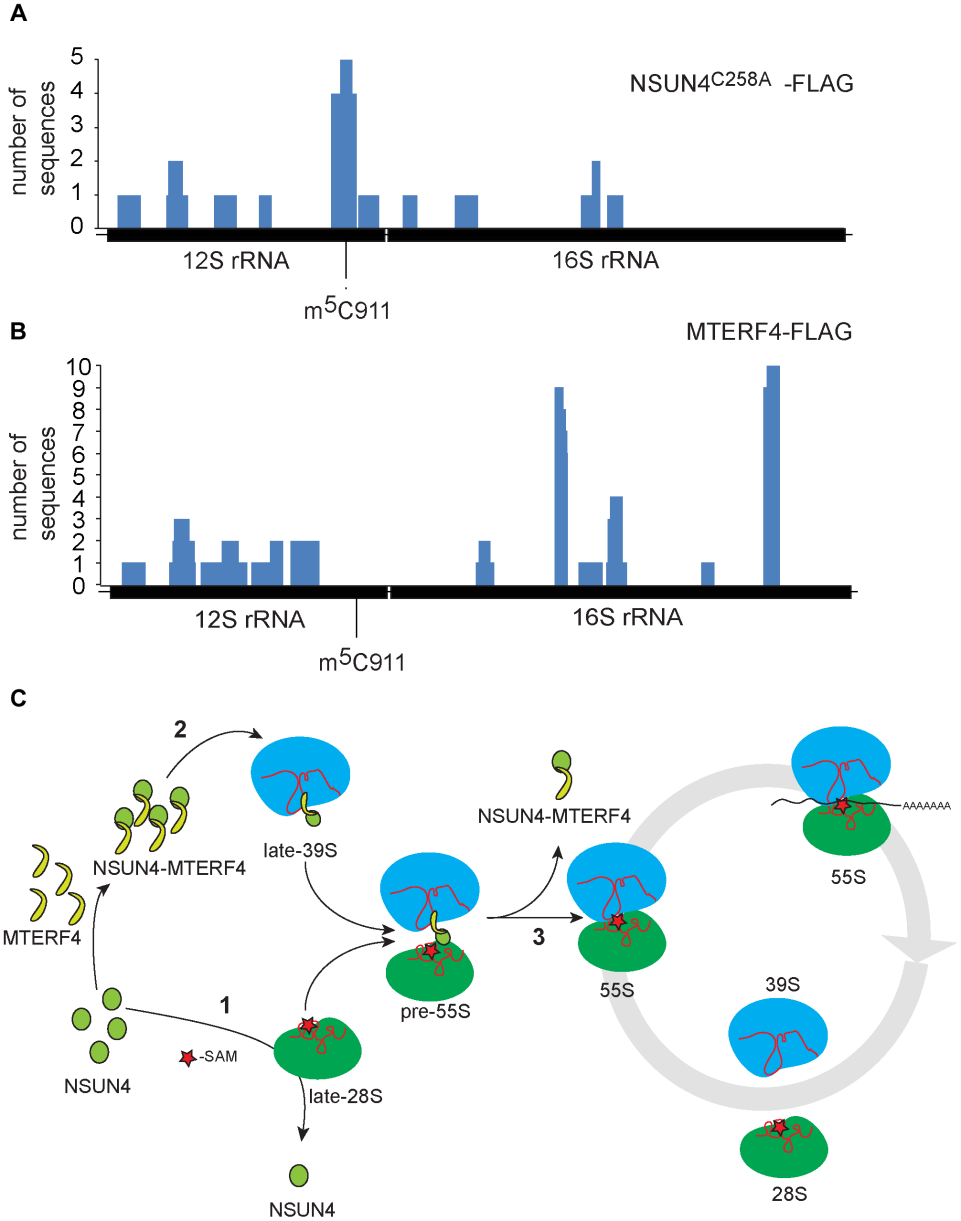
rRNA binding by NSUN4 and MTERF4 and model of the role of the NSUN4/MTERF4 complex in regulation of ribosome assembly. A. Location, along mtDNA, of the RNA fragments identified after CLIP analysis on HeLa cells expressing “trap” mutant of NSUN4, NSUN4^C258A^-FLAG. B. Location, along mtDNA, of the RNA fragments identified after PAR-CLIP analysis on HeLa cells expressing MTERF4-FLAG. C. Model for the role of NSUN4 in mitoribosomal assembly. (1) NSUN4 methylates 12S rRNA. Red star denotes the methyl group donated by SAM. (2) The NSUN4/MTERF4 complex is incorporated into the LSU. (3) Release of the NSUN4/MTERF4 complex from LSU enables the interaction between both subunits.

We performed photoactivatable ribonucleoside-enhanded cross linking and immunoprecipitation (PAR-CLIP) experiments in HeLa cells expressing either MTERF4-FLAG or NSUN4-FLAG to identify interacting regions on 16S and 12S rRNA. Using this approach, we were able to detect RNA fragments specifically cross-linked to regions of 12S and 16S rRNA. The number of interacting RNA fragments was small, which likely reflects the fact that only a small amount of NSUN4/MTERF4 is associated with the LSU under normal conditions [8]. This is expected as NSUN4/MTERF4 is involved in specific transient steps of ribosomal biogenesis and therefore is unlikely to be associated with every mature mitochondrial ribosome. Additionally, we performed CLIP experiments using cells expressing a trap-mutant of NSUN4 (NSUN4^C258A^-FLAG), which is able to form a covalent crosslink with the RNA substrate subjected to site-specific methylation [24], [25]. With this latter approach we identified 16 RNA fragments mapping to 12S rRNA (Figure 5A and Table S1). Five of these fragments of different lengths encompassed the region containing C911. The remaining sequences were distributed along 12S or 16S rRNA and likely represent additional specific or unspecific interactions with the fully assembled ribosome. Finally, MTERF4-FLAG was reproducibly found to be crosslinked to two 16S rRNA regions in two independent PAR-CLIP experiments (Figure 5B and Table S2). This finding is rather unexpected and suggests that the MTERF4/NSUN4 complex may interact with two different LSU regions simultaneously. Unfortunately, there is no atomic resolution structure the mitochondrial ribosome and we can therefore not exactly pinpoint the location of these regions. The RNA sequences identified by the CLIP experiments are predicted to form mixed double- and single-stranded structures [26] and we therefore tested whether the MTERF4/NSUN4 complex has any structural preferences when binding RNA. We performed gel shift experiments with short RNA fragments incubated with the recombinant NSUN4/MTERF4 complex or the recombinant NSUN4 protein. The NSUN4/MTERF4 complex binds RNA fragments with a pronounced double-stranded conformation (Figure S5A; right panels), whereas it poorly binds single-stranded RNA fragments derived from the same double-stranded RNA fragment (Figure S5A; left panels). These findings indicate that NSUN4/MTERF4 preferentially binds double-stranded RNA. In view of our finding that NSUN4 is able to methylate C911 independent of its interaction with MTERF4, we also tested if NSUN4 directly can bind an RNA fragment (ds12S: 878–949) containing the methylation site and indeed found such a specific interaction (Figure S5B), showing that NSUN4 can bind RNA on its own in the absence of MTERF4.

## Discussion

We demonstrate here that NSUN4 unexpectedly is a dual function protein. On the one hand, NSUN4 can alone methylate C911 in 12S rRNA and on the other hand NSUN4 in complex with MTERF4 is targeted to the LSU to regulate mitoribosomal assembly. The role for NSUN4 in rRNA modification can be uncoupled from its role in mitoribosomal assembly as *Mterf4* knockout mice cannot assemble functional ribosomes despite the presence of the methylated C911 residue. Also other methyltransferases have been suggested to have dual functions. The yeast methyltransferase, Dim1p, which generates the highly conserved m^6^_2_Am^6^_2_A on cytoplasmic SSU rRNA, also has a role in rRNA processing [27]. With the use of different *dim1* alleles both functions can be uncoupled, revealing that dimethylation is dispensable for growth whereas the role in rRNA processing is essential [28]. The bacterial ortholog of Dim1p, KsgA, has also been suggested to have dual roles in SSU biogenesis by establishing the m^6^_2_Am^6^_2_A modification of the SSU rRNA and by sterically blocking access for binding of the LSU and the initiation factor 3 to regulate the formation of assembled bacterial ribosomes [29], [30]. Moreover, conformational changes upon binding of the methyltransferase RsmC to its substrate, G1207, were proposed to protect rRNA against misfolding during SSU assembly [31]. Similar to the examples above, we hypothesize that the NSUN4/MTERF4 methylation complex plays a critical structural role during LSU assembly through its affinity for double-stranded rRNA. The NSUN4/MTERF4 complex, when bound to the LSU, may inhibit the formation of assembled ribosomes by two possible, but not necessarily mutually exclusive, mechanisms: i) by physically preventing interaction between both ribosomal subunits and ii) by occluding a putative 16S rRNA fragment required for subunit interaction or LSU activation. Ribosomal assembly in bacteria is characterized by continuous structural rearrangements of rRNA, which sometimes transit through misfolded states stabilized through interactions with ribosomal proteins or ribosomal assembly factors [32]. Later, these states are resolved through refolding of rRNA, release of the ribosomal assembly factors or both. It is possible that release of the NSUN4/MTERF4 complex results in conformational changes in 16S rRNA, which in turn can activate the LSU to enable and stabilize its interaction with the SSU. Thus the NSUN4/MTERF4 complex may also play an important structural role by preventing premature entry into translation.

The exact mechanistic function of m^5^-methylation on cytosine in RNA is generally unclear. An m^5^-methylation does not impair base pairing nor does it appear to induce conformational changes in the modified nucleotide. However, studies on tRNA^Phe^ and tRNA^Val(AAC)^ have suggested that m^5^C, likely in cooperation with other modified residues, is involved in the stabilization of tRNA folding under physiological conditions [33]–[35]. Similarly m^5^C911 may cooperate with the nearby m^4^C909 and other rRNA modifications in stabilization of 12S rRNA folding, thereby facilitating mitoribosomal assembly. Detailed crystallographic analyses on mitoribosomal structure are lacking, which hinders a detailed understanding of the importance of C911 for mitoribosomal assembly.

Based on the analyses of our mutant mouse strains, we have attempted to build a preliminary model for the late stages of mitoribosomal subunit assembly and the formation of translation competent assembled ribosomes (Figure 5C). According to this model, NSUN4 methylates C911 in 12S rRNA of SSU (Figure 5C: step 1). This step does not require interaction with MTERF4, suggesting that NSUN4 may be targeted by either interacting directly with the rRNA substrate or by interacting with an unknown SSU protein. Other modifications, like m^6^_2_A1006 and m^6^_2_A1007 by TFB1M, are established independently of m^5^C911 and stabilize the newly formed SSUs. NSUN4/MTERF4 complexes are incorporated into the LSU during its assembly (Figure 5C: step 2). LSU-bound NSUN4/MTERF4 complexes prevent partially assembled LSUs from forming abortive 55S complexes with available SSUs. Finally, LSU-bound NSUN4/MTERF4 complexes are dissociated from the LSU to enable its interaction with the SSU to form translation-competent ribosomes (Figure 5C: step 3).

In conclusion, we show here that NSUN4 is a dual function protein involved in coordinating ribosomal biogenesis in mammalian mitochondria by methylation C911 on the 12S rRNA and by interacting with MTERF4 to regulate the assembly of the two ribosomal subunits. We propose that NSUN4 functions as a quality control step late in ribosomal biogenesis to ensure that only mature SSUs and LSUs are assembled into functional mitoribosomes.

## Methods

### Ethics statement

This study was performed in strict accordance with the recommendations and guidelines of the Federation of European Laboratory Animal Science Associations (FELASA). The protocol was approved by the “Landesamt für Natur, Umwelt und Verbraucherschutz Nordrhein-Westfalen”.

### Generation of Nsun4 knockout mice

The *Nsun4^loxP/+^* mice, that have exon II flanked by loxP-sites, were generated at TaconicArtemis GmbH (Cologne, Germany). The *Nsun4^loxP/+^* mice were mated with mice ubiquitously expressing cre-recombinase to generate heterozygous knockout mice (*Nsun4^+/−^*). Heart- and skeletal muscle-specific knockout mice were generated as described previously [4], [8], [21], [22]. *Nsun4^loxP/loxP^* mice were crossed with transgenic mice expressing cre-recombinase under the control of the muscle creatinine kinase promoter (*Ckmm-cre*). The resulting double heterozygous mice (*Nsun4^loxP/+^*, *+/Ckmm-cre*) were mated with *Nsun4^loxP/loxP^* mice to generate tissue-specific knockout (*Nsun4^loxP/loxP^*, *+/Ckmm-cre*) and control (*Nsun4^loxP/loxP^*) mice.

### Bisulfite mapping of m^5^C residues in mitochondrial rRNA

RNA for bisulfite treatment was isolated using the miRNeasy Mini Kit (QIAGEN) and treated with TURBO DNase (Ambion) to remove mitochondrial DNA. Bisulfite treatment was performed as described previously [34]. Treated RNA was converted to cDNA and sequenced using the Illumina platform. Reads were submitted to fastqc for quality analysis. Fastq files were clipped with fastx_trimmer (fastx toolkit, http://hannonlab.cshl.edu/fastx_toolkit/) to remove the first 4 and last 35 bases, which were composed of lower quality bases at the start and end of the sequences. Clipped paired reads were aligned to the mouse reference genome with the Bismark software [36]. Bismark performs bisulfite mapping and allows calculation of methylation rates via perl scripts. Bisulfite reads are transformed into a C-to-T and G-to-A version (reverse strand) which are aligned to equivalently pre-converted forms of the reference genome using four parallel instances of the Bowtie aligner [37]. Duplicate sequences were removed and data were reevaluated. Methylation rates were calculated using Perl scripts from the Bismark website, (http://www.bioinformatics.babraham.ac.uk /projects/bismark/).

### BN-PAGE and in organello experiments

BN-PAGE experiments were performed using NativePAGE Novex Bis-Tris Gel System (Invitrogen) according to the manufacturer's recommendations. Heart mitochondria (40 µg) were solubilized with NativePAGE Sample Buffer containing 1% dodecylmaltoside (DDM). After 20 minutes on ice, samples were centrifuged (30 min, 16000×g, 4°C). Supernatants were supplemented with NativePAGE G250 Sample Additive and fractionated through 4–16% NativePAGE Novex Bis-Tris Gel. Respiratory chain complexes were transferred onto PVDF membrane and detected using specific antibodies. Pulse labeling of mitochondrial transcription products was performed in isolated mitochondria according to [38], [39]. In organello translation was performed as previously described [22], [40].

### Linear-density sucrose gradients

Assembly of 28S and 39S ribosomal subunits as well as 55S monosomes was assayed using ultracentrifugation through a 10–30% linear-density sucrose gradient as described previously [22].

### Quantification of mitochondrial DNA using TaqMan RT-PCR

For quantification of mtDNA, total DNA was isolated from heart tissue using the DNeasy Blood & Tissue Kit (QIAGEN). Semiquantitative RT-PCR was carried out on 4 ng of total DNA in a 7900HT Real Time PCR system (Applied Biosystems), using TaqMan probes specific for the CoxI and 18S genes (Applied Biosystems).

### Gel shift assays

Purification of human recombinant NSUN4/MTERF4 complex for gel shift experiments was done according to [9]. RNA fragments were purchased from Thermo Scientific or Eurofins MWG Operon. Gel shift experiments were carried out using varying quantities of NSUN4/MTERF4, and in the presence of 40 ng RNA essentially as described in [15]. Fragment ds12S:878–949, which carries C911 in human 12S rRNA, contains three G and three C residues at the 5′- and 3′-end, respectively, to prevent formation of single-stranded termini. See Table S3 for sequences of the RNA fragments used for gel shift experiments.

#### Primer extension

Primer extensions were performed on total RNA from heart tissue as described previously [4]. Samples were analyzed by size fractionation through a 6% polyacrylamide-7M urea gel and subjected to autoradiography.

### Nucleic acid sequence alignments

Sequence alignments were performed using Clustal Omega [41] at default settings and were visualized using GeneDoc at shade level 1 [42]. Sequence accession numbers are given in figure legends.

### Northern blot analysis

RNA for northern blot analysis was isolated using Trizol Reagent (Invitrogen) and resuspended in formamide (Ambion). For detection of mitochondrial transcripts, 1–2 µg of total RNA was denatured in NorthernMax-Gly Sample Loading Dye (Ambion), separated in 1.2% agarose gels containing formaldehyde (SIGMA-Aldrich) and transferred to Hybond-N+ membranes (GE Healthcare). DNA probes, for the detection of mitochondrial mRNAs and rRNAs, were radiolabeled with α-^32^P-dCTP using the Prime-It II random primer labeling kit (Stratagene). For detection of tRNAs, oligonucleotides were labeled with γ-^32^P-ATP using T4-polynucleotide kinase (NEB).

### Western blot analysis

Rabbit polyclonal antisera were used for the detection of TFAM, ATP8 and COXII [43]. A rabbit polyclonal antiserum was used for detection of LRPPRC [22]. Affinity purified rabbit polyclonal antibodies were used for the detection of TFB2M [4], MRPS15 [4] and MRPL13 [8]. Monoclonal antibodies against VDAC were purchased from Calbiochem. Immunodetection of NDUFA9, SDHA, UQCRC2, COX IV and ATP5A1 was performed with monoclonal antibodies from MitoSciences. Monoclonal antibodies against mouse NSUN4 were generated by AbD Serotec.

### DNA constructs and cell lines

The HeLa cells stably transfected with pTreTight-h*Mterf4-FLAG* DNA construct are described previously [8]. HeLa cell clones stably transfected with pTreTight-h*Nsun4-FLAG* and pTreTight-h*NSUN4^C258A^-FLAG* were generated as described [8].

### Crosslinking Immunoprecipitation (CLIP)

Photoactivatable-Ribonucleoside-Enhanced Crosslinking and Immunoprecipitation (PAR-CLIP) was performed by combining and adapting previously described methods [44], [45]. HeLa cells transfected with doxycycline-inducible *MTERF4-FLAG*, *NSUN4-FLAG* and *NSUN4^C258A^-FLAG* containing DNA constructs were induced with 1 µg/ml doxycycline and grown in the presence of 4-thiouridine (4-SU, final concentration of 100 µM) for 14 hours. The cells from 20 culture dishes (500 cm^2^ each) were washed with PBS, crosslinked at 365 nm, collected and frozen in liquid nitrogen. Next, cells were lysed, treated with RNase T1 and used for immunoprecipitation experiments with ANTI-FLAG M2 magnetic beads according to the manufacturer's recommendations (Sigma). The samples were further processed according to [44].

## Supporting Information

Figure S1 Steady-state levels of mtDNA and mitochondrial transcripts. A. Steady-state levels of mtDNA in hearts from 20 weeks-old control (L/L; n = 3) and knockout (L/L, cre; n = 3) mice. mtDNA was detected using a *CoxI*-specific TaqMan probe. 18S rDNA was used as a loading control. Data are represented as mean +/− SEM. ***p<0.001. Student's t test. B. Northern blot analysis of the steady-state levels of mitochondrially encoded rRNAs, mRNAs and tRNAs from 20 weeks-old control (L/L) and mutant (L/L, cre) mice. Nucleus-encoded 18S rRNA is used as a loading control. C. Quantification of the steady-state levels of rRNA, mRNA and tRNA detected by autoradiography. For rRNA and mRNA: number of analyzed animals at 5 weeks, L/L n = 3; L/L, cre n = 3; at 10 weeks, L/L n = 4; L/L, cre n = 4; at 15 weeks, L/L n = 4; L/L, cre n = 4; 20 weeks, L/L, n = 4; L/L, cre n = 4. For tRNA: number of analyzed animals at 20 weeks, L/L, n = 4; L/L, cre n = 4. Data are represented as mean +/− SEM. *p<0.05; **p<0.01; ***p<0.001. Student's t test.
(TIF)

Figure S2 *De novo* transcription in *Nsun4* knockout hearts. A. Analysis of mitochondrial transcription after pulse labeling in isolated heart mitochondria from 20 weeks-old control (L/L) and knockout mice (L/L, cre). B. Western blot analysis to determine steady-state levels of TFB2M and TFAM in mitochondrial extracts from control (L/L) and knockout (L/L, cre) mice at 5 and 20 weeks of age. C. Western blot analysis of LRPPRC levels in mitochondrial extracts from control (L/L) and knockout (L/L, cre) mice at 5, 10, 15 and 20 weeks of age.
(TIF)

Figure S3 Sequence alignments and analysis of adenine dimethylation in 12S rRNA. A. Nucleotide sequence alignment of the rRNA of the SSU containing the m4C and m5C modification in hamster (*M. aureus*, NC_013276.1), mouse (*M.musculus*, NC_005089.1) and human (*H.sapiens*, NC_012920.1). The location of m4C and m5C is indicated. Nucleotide numbering is from the 5′-end of tRNA^Phe^. B. Primer extension analysis of 12S rRNA from control (L/L) and tissue-specific *Nsun4* knockout mice (L/L, cre). Autoradiography of samples separated in a polyacrylamide-urea gel is shown. Primer extension with an oligonucleotide annealing close to the 5′-end of 12S rRNA gives rise to the extension product P1, which is used as a loading control. Dimethylation of 12S rRNA at A1006 and A1007 case a partial stop of the primer extension reaction and thereby generates the extension product P2. Unextended primers are indicated in the bottom of the figure. C. Quantification of the ratio of primer extension products P2 to P1 (see panel A) in control (L/L, n = 4) and tissue-specific *Nsun4* knockout (L/L, cre n = 4) mice. Data represent mean +/− SEM.
(TIF)

Figure S4 C911 methylation rate in *Mterf3*-knockout mice. Relative methylation rate of 12S rRNA in control (N = 1) and *Mterf3* knockout (N = 2) hearts at 14 weeks of age.
(TIF)

Figure S5 rRNA binding by NSUN4 and NSUN4/MTERF4 complex tested by EMSA. A. Gel shift assays to determine binding of the recombinant NSUN4/MTERF4 complex to ssRNA and dsRNA of 16S rRNA. Filled triangles denote increasing concentrations of recombinant proteins: 0, 0.02, 0.04, 0.08, 0.16, 0.32, 0.64, 1.28, 2.56 µM. Nucleotide numbering is relative to the 5′-end of the human mitochondrial gene for tRNA^Phe^. ss, single-stranded; ds, double-stranded (first row). B. Gel shift assays to determine binding of the recombinant NSUN4 to a double-stranded fragment from 12 rRNA containing the methylation substrate C911. Analysis was performed as in A.
(TIF)

Table S1 Sequences of the RNA fragments identified after CLIP experiments performed on HeLa cells expressing NSUN4^C258A^-FLAG. Positions of the RNA fragments along mtDNA relative to the beginning of *tRNA^Phe^* are indicated. C911 is indicated in red.
(DOC)

Table S2 Sequences of the RNA fragments identified after PAR-CLIP experiments performed on HeLa cells expressing MTERF4-FLAG. Positions of the RNA fragments along mtDNA relative to the beginning of *tRNA^Phe^* are indicated. Results from two independent experiments were pooled.
(DOC)

Table S3 Sequences of the RNA fragments used for gel shift experiments. Positions and sequences of the RNA fragments used for the gel shift experiments are listed in the table.
(DOC)
