# Supplementary figures and images for: NSUN4 Is a Dual Function Mitochondrial Protein Required for Both Methylation of 12S rRNA and Coordination of Mitoribosomal Assembly
Source: PLoS Genet. 2014 Feb 6;10(2):e1004110. doi: 10.1371/journal.pgen.1004110 (PMC3916286; doi:10.1371/journal.pgen.1004110)

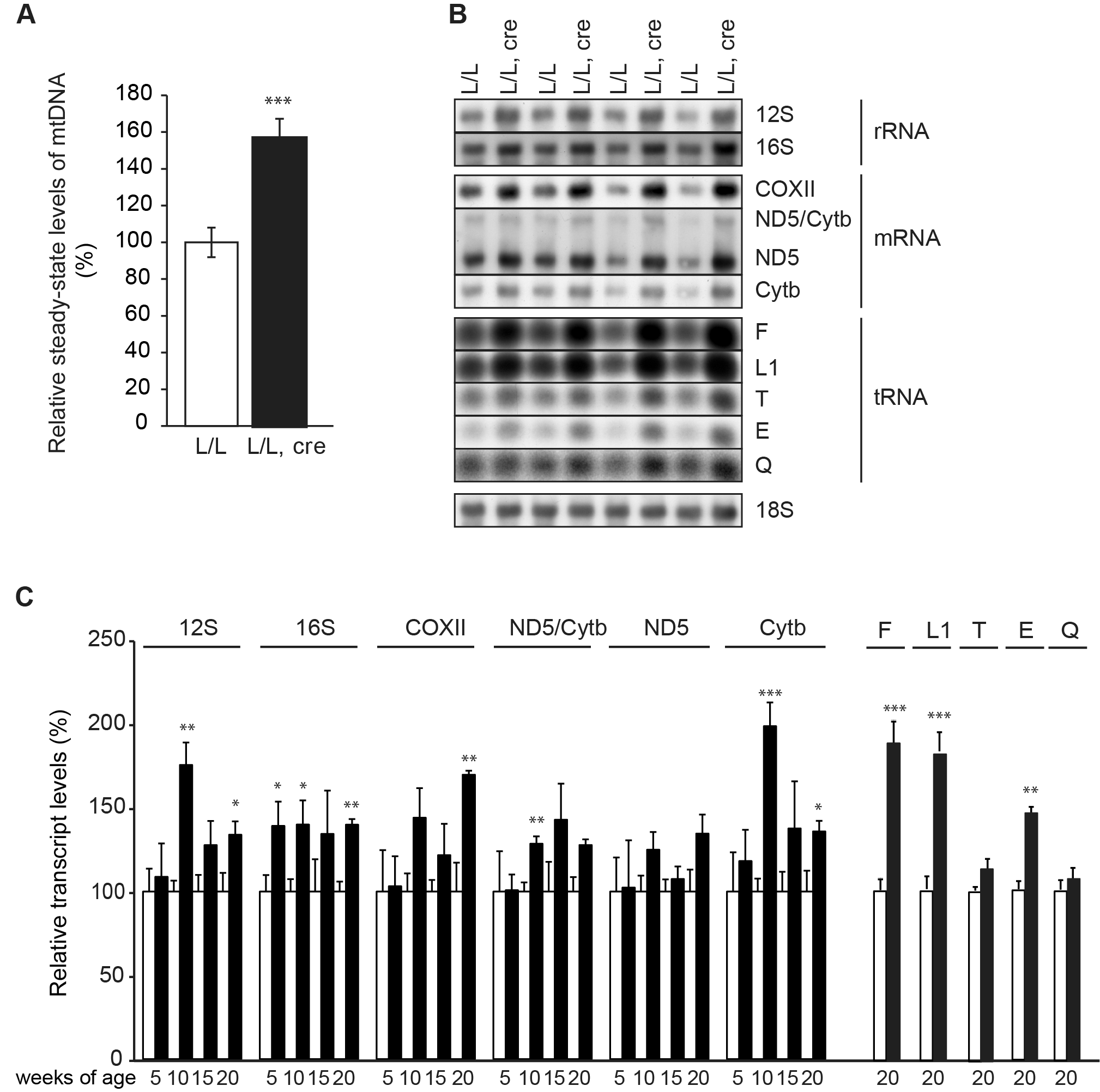

Supplement: Figure S1 — Steady-state levels of mtDNA and mitochondrial transcripts. A. Steady-state levels of mtDNA in hearts from 20 weeks-old control (L/L; n = 3) and knockout (L/L, cre; n = 3) mice. mtDNA was detected using a CoxI-specific TaqMan probe. 18S rDNA was used as a loading control. Data are represented as mean +/− SEM. ***p<0.001. Student's t test. B. Northern blot analysis of the steady-state levels of mitochondrially encoded rRNAs, mRNAs and tRNAs from 20 weeks-old control (L/L) and mutant (L/L, cre) mice. Nucleus-encoded 18S rRNA is used as a loading control. C. Quantification of the steady-state levels of rRNA, mRNA and tRNA detected by autoradiography. For rRNA and mRNA: number of analyzed animals at 5 weeks, L/L n = 3; L/L, cre n = 3; at 10 weeks, L/L n = 4; L/L, cre n = 4; at 15 weeks, L/L n = 4; L/L, cre n = 4; 20 weeks, L/L, n = 4; L/L, cre n = 4. For tRNA: number of analyzed animals at 20 weeks, L/L, n = 4; L/L, cre n = 4. Data are represented as mean +/− SEM. *p<0.05; **p<0.01; ***p<0.001. Student's t test. (TIF) [file pgen.1004110.s001.tif]

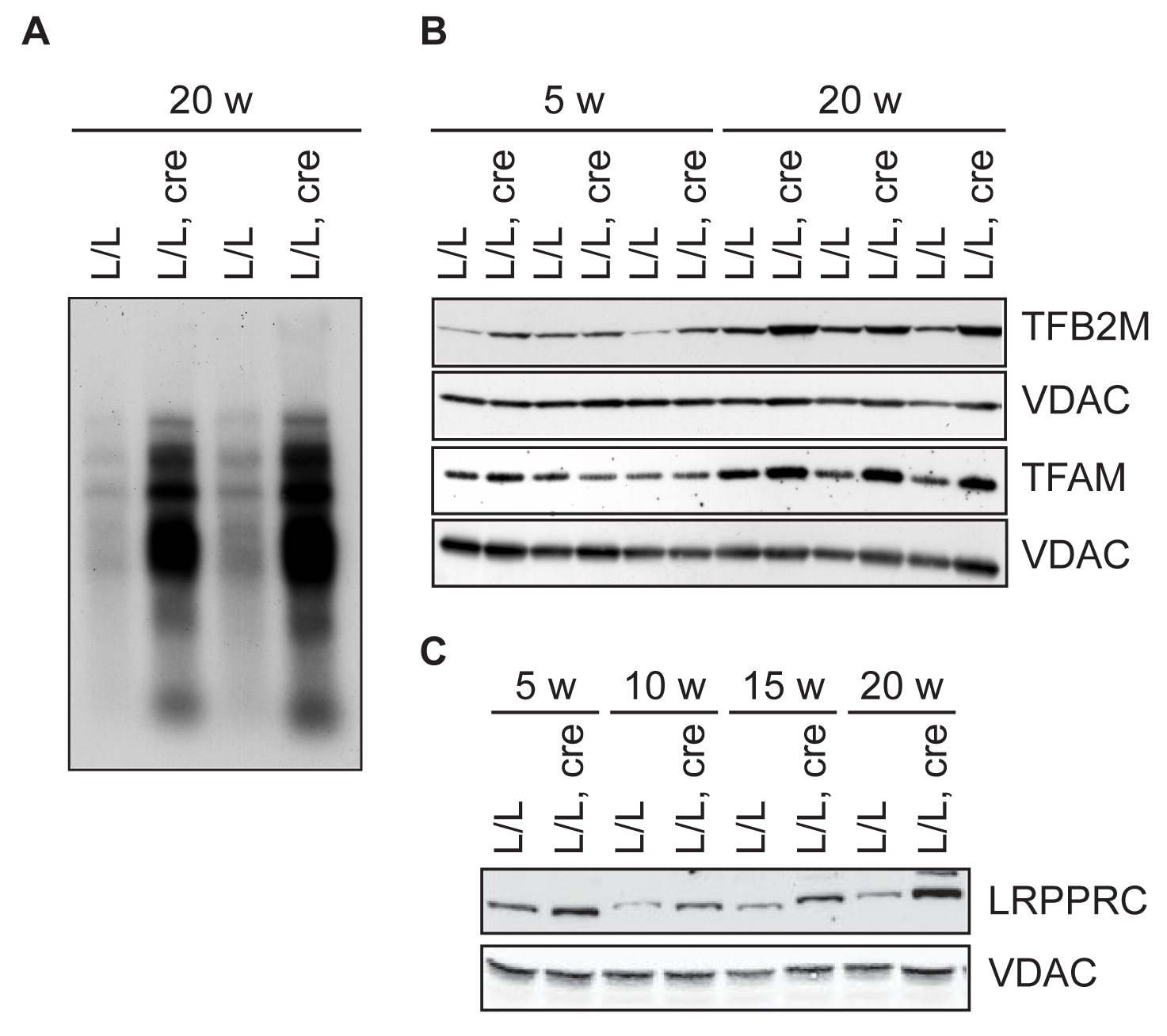

Supplement: Figure S2 — De novo transcription in Nsun4 knockout hearts. A. Analysis of mitochondrial transcription after pulse labeling in isolated heart mitochondria from 20 weeks-old control (L/L) and knockout mice (L/L, cre). B. Western blot analysis to determine steady-state levels of TFB2M and TFAM in mitochondrial extracts from control (L/L) and knockout (L/L, cre) mice at 5 and 20 weeks of age. C. Western blot analysis of LRPPRC levels in mitochondrial extracts from control (L/L) and knockout (L/L, cre) mice at 5, 10, 15 and 20 weeks of age. (TIF) [file pgen.1004110.s002.tif]

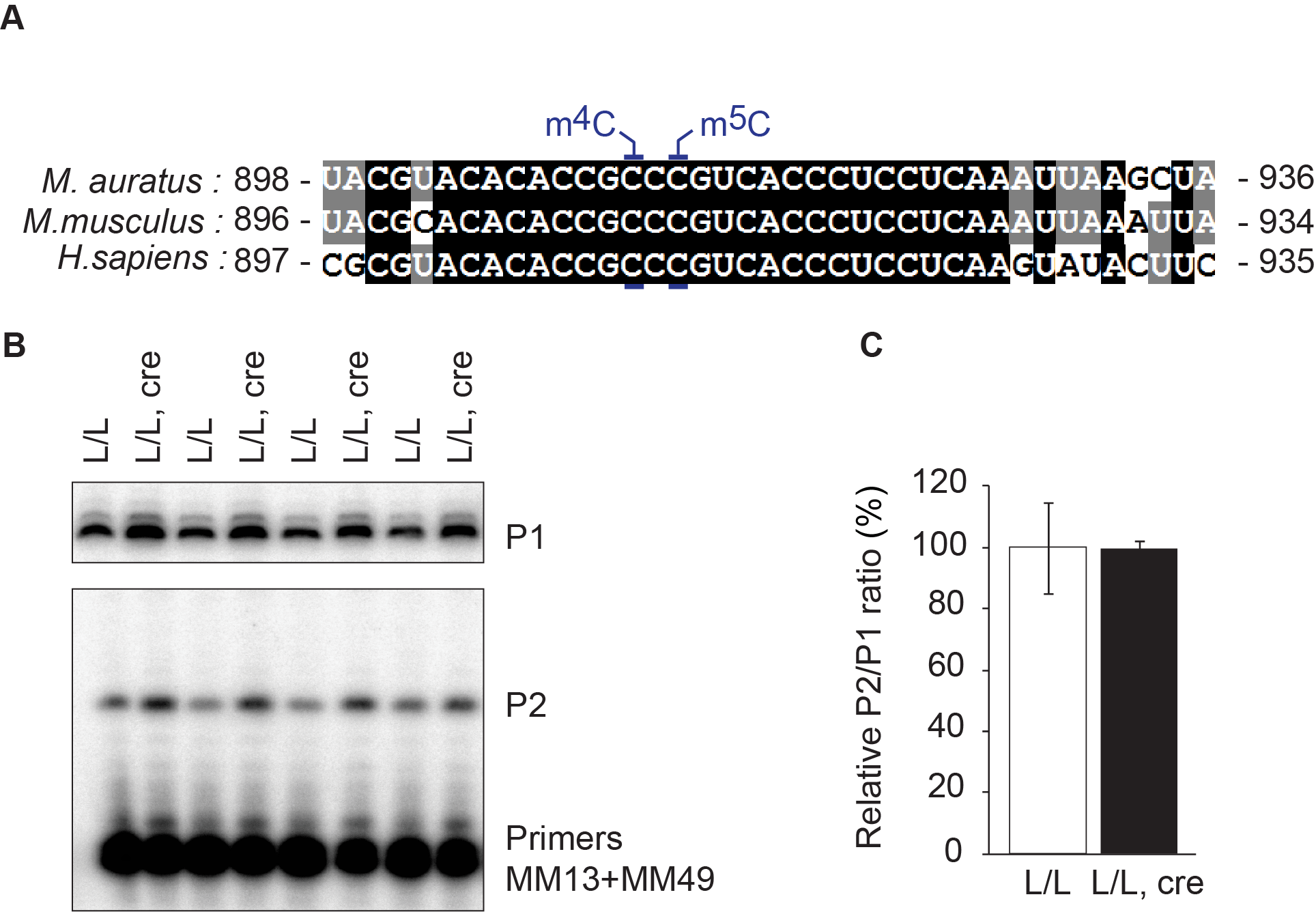

Supplement: Figure S3 — Sequence alignments and analysis of adenine dimethylation in 12S rRNA. A. Nucleotide sequence alignment of the rRNA of the SSU containing the m4C and m5C modification in hamster (M. aureus, NC_013276.1), mouse (M.musculus, NC_005089.1) and human (H.sapiens, NC_012920.1). The location of m4C and m5C is indicated. Nucleotide numbering is from the 5′-end of tRNAPhe. B. Primer extension analysis of 12S rRNA from control (L/L) and tissue-specific Nsun4 knockout mice (L/L, cre). Autoradiography of samples separated in a polyacrylamide-urea gel is shown. Primer extension with an oligonucleotide annealing close to the 5′-end of 12S rRNA gives rise to the extension product P1, which is used as a loading control. Dimethylation of 12S rRNA at A1006 and A1007 case a partial stop of the primer extension reaction and thereby generates the extension product P2. Unextended primers are indicated in the bottom of the figure. C. Quantification of the ratio of primer extension products P2 to P1 (see panel A) in control (L/L, n = 4) and tissue-specific Nsun4 knockout (L/L, cre n = 4) mice. Data represent mean +/− SEM. (TIF) [file pgen.1004110.s003.tif]

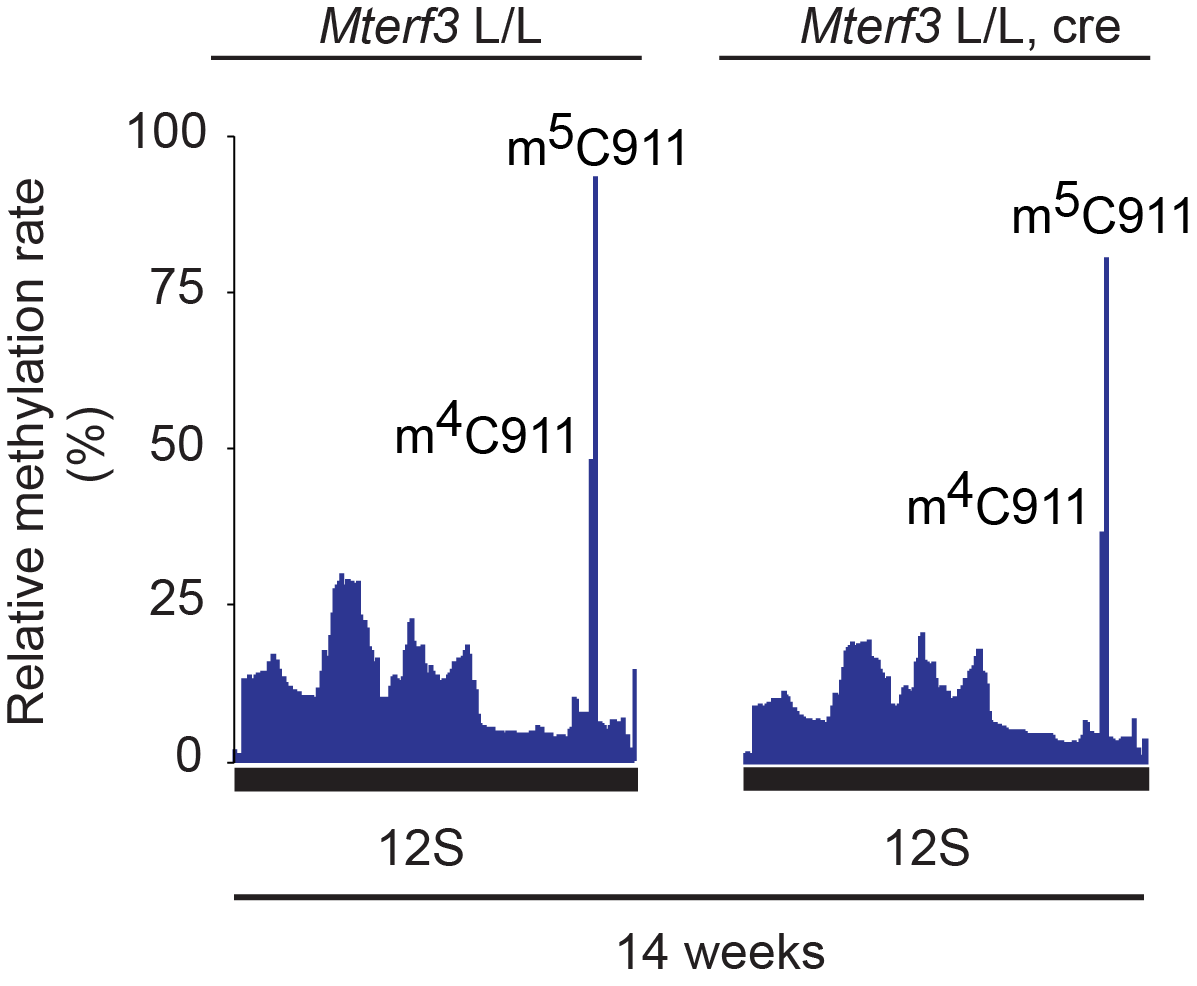

Supplement: Figure S4 — C911 methylation rate in Mterf3-knockout mice. Relative methylation rate of 12S rRNA in control (N = 1) and Mterf3 knockout (N = 2) hearts at 14 weeks of age. (TIF) [file pgen.1004110.s004.tif]

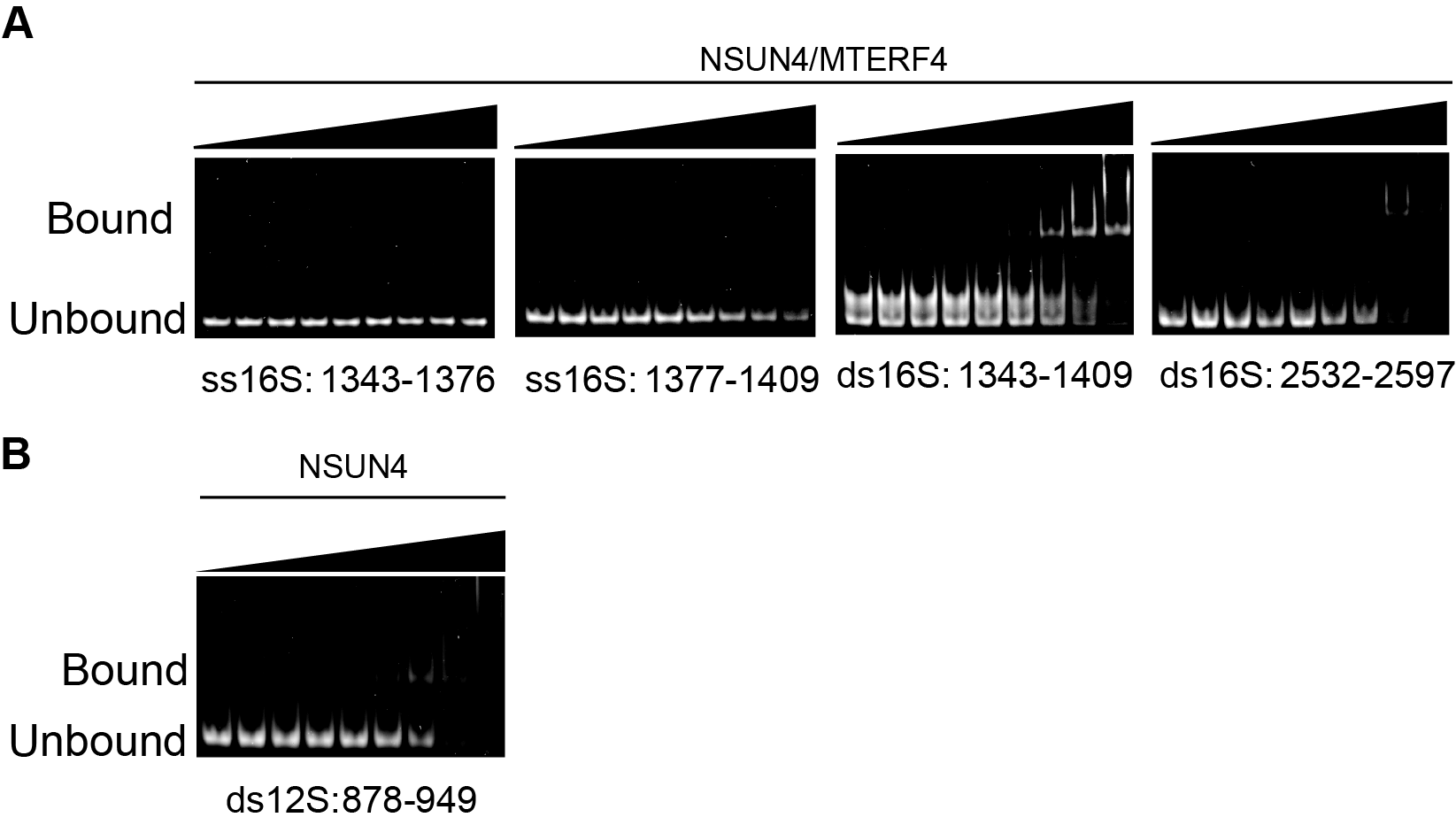

Supplement: Figure S5 — rRNA binding by NSUN4 and NSUN4/MTERF4 complex tested by EMSA. A. Gel shift assays to determine binding of the recombinant NSUN4/MTERF4 complex to ssRNA and dsRNA of 16S rRNA. Filled triangles denote increasing concentrations of recombinant proteins: 0, 0.02, 0.04, 0.08, 0.16, 0.32, 0.64, 1.28, 2.56 µM. Nucleotide numbering is relative to the 5′-end of the human mitochondrial gene for tRNAPhe. ss, single-stranded; ds, double-stranded (first row). B. Gel shift assays to determine binding of the recombinant NSUN4 to a double-stranded fragment from 12 rRNA containing the methylation substrate C911. Analysis was performed as in A. (TIF) [file pgen.1004110.s005.tif]
