# Supplementary material for: NSUN4 Is a Dual Function Mitochondrial Protein Required for Both Methylation of 12S rRNA and Coordination of Mitoribosomal Assembly
Source: PLoS Genet. 2014 Feb 6;10(2):e1004110. doi: 10.1371/journal.pgen.1004110 (PMC3916286; doi:10.1371/journal.pgen.1004110)
Supplement: Table S1 — Sequences of the RNA fragments identified after CLIP experiments performed on HeLa cells expressing NSUN4C258A-FLAG. Positions of the RNA fragments along mtDNA relative to the beginning of tRNAPhe are indicated. C911 is indicated in red. (DOC) [file pgen.1004110.s006.doc]

| CLIP NSUN4^C258A^-FLAG | | | | |
| --- | --- | --- | --- | --- |
| transcript | start | sequence | end | Number of sequences |
| 12S | 73 | ATAGGTTTGGTCCTAGCCTTTCTATTAGCTCTTAGTAAGATTACACATGCAAGCATCCCGTTCCAGTGAGTTCACCCTC | 153 | 1 |
| 12S | 246 | GATTAACCTTTAGCAATAAACGAAAGTTTAACTAAGCTATACTAACCCCAGGGTTGG | 303 | 1 |
| 12S | 253 | CTTTAGCAATAAACGAAAGTTTAACTAAGCTATACTAACCCCAGGGTTGGTCAATTTCGGCCAGCCACC | 323 | 1 |
| 12S | 417 | AAAAAACTCCAGTTGACACAAAATAGACTACGAAAGTGGCTTTAACATATCTGAACACA AATAGCTAAGACCCAAACTGG | 498 | 1 |
| 12S | 579 | GCTTAAAACTCAAAGGACCTGGCGGTGCTTCATATCCCTCTA | 621 | 1 |
| 12S | 839 | GAAGGTGGATTTAGCAGTAAACTAAGAGTAGAGTGCTTAGTTGAACAGGGCCCTGAAGCCGTACACACCGC**C**CGTCACCCTCCTCAA | 927 | 4 |
| 12S | 869 | GAGTGCTTAGTTGAACAGGGCCCTGAAGCGCGTACACACCGC**C**CG | 913 | 1 |
| 12S | 935 | CAAAGGACATTTAACTAAAACCCCTACGCATTTATATAGAGGAGACAAGTCGTAACATGGTAAGTGTACTGG | 1006 | 1 |
| 12S | 1095 | GCTAAACCTAGCCCCAAACCCACTCCACCTTACTACCAGACAACCTTA | 1143 | 1 |
|  |  |  |  |  |
| 16S | 1282 | GAATTAACTAGAAATAACTTTGCAAGGAGAGCCAAAGCTAAGACCCCCGAAACCAGACGGCTACCTAAGAACAGCTAAAA | 1363 | 1 |
| 16S | 1736 | AACATGAAAACATTCTCCTCCGCATAAGCCTGCGTCAGATTAAAACACTGAACTGACAATAACA | 1801 | 1 |
| 16S | 1773 | ATTAAAACACTGAACTGACAATTAACA | 1801 | 1 |
| 16S | 1827 | GTCATTATTACCCTCACTGTCAACCCAACACAGGCATGCTCATAAGGAAAGGTTAAA | 1883 | 1 |

**Table S1: Sequences of the RNA fragments identified after CLIP experiments performed on HeLa cells expressing NSUN4^C258A^-FLAG.**
