# Supplementary material for: NSUN4 Is a Dual Function Mitochondrial Protein Required for Both Methylation of 12S rRNA and Coordination of Mitoribosomal Assembly
Source: PLoS Genet. 2014 Feb 6;10(2):e1004110. doi: 10.1371/journal.pgen.1004110 (PMC3916286; doi:10.1371/journal.pgen.1004110)
Supplement: Table S2 — Sequences of the RNA fragments identified after PAR-CLIP experiments performed on HeLa cells expressing MTERF4-FLAG. Positions of the RNA fragments along mtDNA relative to the beginning of tRNAPhe are indicated. Results from two independent experiments were pooled. (DOC) [file pgen.1004110.s007.doc]

**Table S2: Sequences of the RNA fragments identified after PAR-CLIP experiments performed on HeLa cells expressing MTERF4-FLAG.**

| PAR-CLIP MTERF4-FLAG | | | | |
| --- | --- | --- | --- | --- |
| transcript | start | sequence | end | Number of sequences |
| 12S | 236 | AAACAGCAGTGATTAACCTTTAGCAATAAACGAAAGTTTAACTAAGCTATACTAACCCCAGGGTTGGTCAATTTCGTGCCAGCCAC | 321 | 1 |
| 12S | 344 | GTCAATAGAAGCCGGCGTAAAGAGTGTTTTAGATCACCCCCTCCCCAATAAAGCTAAAACTCACCTGAGTTG | 415 | 1 |
| 12S | 415 | GTAAAAAACTCCAGTTGACACAAAATAGACTACGAAAGTGGCTTTAACATATCTG | 469 | 1 |
| 12S | 516 | GCTTAGCCCTAAACCTCAACAGTTAAATCAACAAAACTGCTCGCCAGAACACTACGAGCACAGCTTAAAACTCAAAGGACCTGGCGGTGCTTCATATCCCTCTA | 621 | 1 |
| 12S | 579 | GCTTAAAACTCAAAGGACCTGGCGGTGCTTCATATCCCTCTAG | 621 | 1 |
| 12S | 651 | GATCAACCTCACCACCTCTTGCTCAGCCTATATACCGCCATCTTCAGCAAACCCTGATGAGGCTACAAAGTAAGCGCAAGTACCCACGTAAA | 744 | 2 |
|  |  |  |  |  |
| 16S | 1283 | AATTAACTAGAAATAACTTTGCAAGGAGAGCCAAAGCTAAGACCCCCGAAACCAGACGAGC | 1343 | 1 |
| 16S | 1292 | GAAATAACTTTGCAAGGAGAGCCAAAGCTAAGACCCCCG | 1330 | 1 |
| 16S | 1552 | GGAAAAAACCTTGTAGAGAGAGTAAAAAATTTAACACCCATA | 1594 | 6 |
| 16S | 1552 | GGAAAAAACCTTGTAGAGAGAGTAAAAAA | 1580 | 1 |
| 16S | 1552 | GGAAAAAACCTTGTAGAGAGAGTAAAAAATTTAAC | 1586 | 1 |
| 16S | 1552 | GGAAAAAACCTTGTAGAGAGAGTAAAAAATTTAACACCCA | 1591 | 1 |
| 16S | 1633 | GCTCAACACCCACTACCTAAAAAATCCCAAACATATAACTGAACTCCTCACACCCAATTGGACCAATCTATCACCCTATA | 1713 | 1 |
| 16S | 1728 | GTATAAGTAACATGAAAACATTCTCCTCCGCATAAGCCTGCGTCAGATTAAAACACTGAACTGACAATTAA | 1798 | 1 |
| 16S | 1741 | GAAAACATTCTCCTCCGCATAAGCCTGCGTCAGATTAAAAC | 1781 | 1 |
| 16S | 1734 | GTAACATGAAAACATTCTCCTCCGCATAAGCCTGCGTCAGATTAAAA | 1780 | 1 |
| 16S | 1734 | GTAACATGAAAACATTCTCCTCCGCATAAGCCTGCGTCAGATTAAAACA | 1782 | 1 |
| 16S | 2055 | GAATGGCTCCACGAGGGTTCAGCTGTCTCTTACTTTTAACCAG | 2097 | 1 |
| 16S | 2267 | CGAGCAGTACATGCTAAGACTTCACCAGTCAAAGCGAACTACTATACTCAATTG | 2320 | 9 |
| 16S | 2278 | TGCTAAGACTTCACCAGTCAAAGCGAACTACTATACTCAATTG | 2320 | 1 |
