# Supplementary material for: NSUN4 Is a Dual Function Mitochondrial Protein Required for Both Methylation of 12S rRNA and Coordination of Mitoribosomal Assembly
Source: PLoS Genet. 2014 Feb 6;10(2):e1004110. doi: 10.1371/journal.pgen.1004110 (PMC3916286; doi:10.1371/journal.pgen.1004110)
Supplement: Table S3 — Sequences of the RNA fragments used for gel shift experiments. Positions and sequences of the RNA fragments used for the gel shift experiments are listed in the table. (DOC) [file pgen.1004110.s008.doc]

**Table S3: Sequences of the RNA fragments used for gel shift experiments**

| **name** | **sequence** |
| --- | --- |
| ss16S:1343-1376 | CUACCUAAGAACAGCUAAAAGAGCACACCCGUCU |
| ss16S:1377-1409 | AUGUAGCAAAAUAGUGGGAAGAUUUAUAGGUAG |
| ds16S:1343-1409 | CUACCUAAGAACAGCUAAAAGAGCACACCCGUCUAUGUAGCAAAAUAGUGGGAAGAUUUAUAGGUAG |
| ds16S:2532-2597 | UUCAAAUUCCUCCCUGUACGAAAGGACAAGAGAAAUAAGGCCUACUUCACAAAGCGCCUUCCCCCG |
| ds12S:878-949 | GGGGUUGAACAGGGCCCUGAAGCGCGUACACACCGCCCGUCACCCUCCUCAAGUAUACUUCAAAGGACAUUUAACCCC |
